# D‐cycloserine‐augmented one‐session treatment of specific phobias in children and adolescents

**DOI:** 10.1002/brb3.984

**Published:** 2018-05-01

**Authors:** Lara J. Farrell, Allison M. Waters, Ella L. Oar, Evelin Tiralongo, Vinay Garbharran, Clair Alston‐Knox, Harry McConnell, Nigel Collings, Melanie Zimmer‐Gembeck, Caroline L. Donovan, Chris Testa, Eric A. Storch, Thomas H. Ollendick

**Affiliations:** ^1^ School of Applied Psychology and Menzies Health Institute QLD Griffith University Southport Qld Australia; ^2^ School of Applied Psychology and Menzies Health Institute QLD Griffith University Mt Gravatt Qld Australia; ^3^ School of Pharmacy and Menzies Health Institute QLD Griffith University Southport Qld Australia; ^4^ Child and Youth Mental Health Services, Queensland Health Southport Qld Australia; ^5^ Griffith Social and Behavioural Research College Griffith University Southport Qld Australia; ^6^ School of Medicine and Menzies Health Institute QLD Griffith University Southport Qld Australia; ^7^ Menzies Health Institute QLD Griffith University Southport Qld Australia; ^8^ Tugun Compounding Pharmacy Tugun Gold Coast Qld Australia; ^9^ Departments of Pediatrics Psychiatry, and Psychology University of South Florida Tampa Florida; ^10^ Rogers Behavioral Health – Tampa Bay Tampa Florida; ^11^ All Children's Hospital – Johns Hopkins Medicine St. Petersburg Florida; ^12^ Department of Psychology Child Study Centre Virginia Polytechnic Institute and State University Blacksburg Virginia

**Keywords:** children, D‐Cycloserine, exposure therapy, one‐session treatment, phobia

## Abstract

**Background:**

D‐Cycloserine has potential to enhance exposure therapy outcomes. The current study presents a preliminary randomized, placebo‐controlled double‐blind pilot trial of DCS‐augmented one‐session treatment (OST) for youth (7–14 years) with specific phobia. A secondary aim of this pilot study was to explore the effects of youth age and within‐session fear reduction as potential moderators of DCS outcomes in order to generate hypotheses for a larger trial. It was hypothesized that DCS would be associated with greater improvements than placebo, that children (7–10 years) would have greater benefits than adolescents (11–14 years), and that DCS effects would be stronger for participants with the greater within‐session fear reduction during the OST.

**Methods:**

Thirty‐five children and adolescents were randomized to either OST combined with DCS (*n *= 17), or OST combined with placebo (PBO;* n *= 18) and assessed at 1 week, 1 month, and 3 month following treatment.

**Results:**

There were no significant pre‐ to post‐treatment or follow‐up benefits of DCS relative to placebo. Secondary analyses of age indicated that relative to PBO, DCS was associated with greater improvements for children (but not adolescents) on measures of severity at 1‐month follow‐up. Children in the DCS condition also showed significantly greater improvement to 1 month on global functioning relative to other groups. Conversely, adolescents had significant post‐treatment benefits in the PBO condition on symptom severity measures relative to DCS, and adolescents in the DCS condition had significantly poorer functioning at 3 months relative to all other groups. Finally, there was a trend for within‐session fear reduction to be associated with moderating effects of DCS, whereby greater reduction in fear was associated with greater functioning at one‐month follow‐up for children who received DCS, relative to PBO.

**Limitations:**

The study sample was small and therefore conclusions are tentative and require replication.

**Conclusions:**

Age and within‐session fear reduction may be important moderators of DCS‐augmented one‐session exposure therapy, which requires testing in a fully powered randomized controlled trial.

## 1 INTRODUCTION

Specific phobias are highly prevalent (Bener, Ghuloum, & Dafeeah, 2011; Kessler et al., 2005; Ollendick, Hagopian, & King, 1997), onset early in life, and in childhood, they are associated with academic disruption (Dweck & Wortman, 1982; Ialongo, Edelsohn, Werthamer‐Larsson, Crockett, & Kellam, 1995; Klein & Last, 1989), social and personal distress (Ollendick & King, 1994; Ollendick, King, & Muris, 2002; Strauss, Lease, Kazdin, Dulcan, & Last, 1989) and interference in daily life (Essau, Conradt, & Petermann, 2000; Ollendick, King, & Muris, 2004; Ollendick et al., 1997). There is strong empirical support (Silverman et al., 1999; Vigerland et al., 2013) for cognitive‐behavioral treatments (CBT) involving exposure therapy, and one variant of CBT, the one‐session treatment (OST) approach developed by Öst (1989), has been deemed *well established* for treating specific phobias (Davis & Ollendick, 2005). However, with as many as 50% of children still experiencing significant symptoms following treatment (Ollendick & Davis, 2013), there remains considerable room for improvement. The potential of novel pharmacological agents to augment exposure‐based therapies has been proposed to be one way to improve such outcomes (Byrne, Farrell, Storch, & Rapee, 2014).

D‐Cycloserine (DCS), a partial glutamatergic N‐methyl‐D‐aspartate (NMDA) agonist, has been found in rodent and human studies to promote both the extinction of conditioned fear and the consolidation of learning associated with extinction training, the theoretical basis of exposure therapy (Davis, Ressler, Rothbaum, & Richardson, 2006). Thus, given that the NMDA receptor is involved in learning and memory processes that underlie fear extinction learning, augmenting OST with DCS may boost treatment effectiveness. In a recent meta‐analysis of DCS trials (Mataix‐Cols et al., 2017), including individual patient‐level data from 21 clinical trials of mostly adults with anxiety disorders, DCS was found to be associated with enhanced positive outcomes from pre‐exposure therapy to post‐treatment (Cohen d = −0.25). Moreover, additional analyses showed that participants assigned to DCS evidenced lower symptom severity than those assigned to placebo at post‐treatment and at follow‐up.

DCS has also shown to boost outcomes for some children with specific phobias. In the only study to date, Byrne et al. (2015) examined DCS‐augmented single‐session exposure therapy in 35 children (6–14 years) with either a dog or spider phobia. They examined generalization of fear extinction by examining postsession fear and avoidance across stimuli and contexts. There were no between‐group differences when a new stimulus was presented in the treatment context at post‐treatment; however, when the new stimulus was presented in a novel context, the DCS group exhibited significantly less fear and avoidance relative to placebo. The authors concluded that children who received DCS achieved greater retention of fear extinction learning, and thus, this learning generalized more readily to novel stimuli across contexts.

### 1.1 Moderators of treatment outcomes for CBT with DCS augmentation

While initial clinical trials of DCS offered promising effects, on a whole, effect sizes across studies are generally small to moderate and they vary, with as many trials with positive results as there are trials with null results. Some of this variation in study effects sizes may be due to heterogeneity in sampling and in study design, including the timing of dose, and number of doses used within each trial. In particular, clinical studies of factors enhancing fear reduction during exposure therapy and neuroscience research on developmental differences in the neural basis of extinction learning suggest that age and within‐session fear reduction may be two important moderators of outcomes of DCS‐augmented exposure therapy for specific phobias in youth.

Studies to date of DCS‐augmented exposure therapy with youth (i.e., Byrne et al., 2015; Farrell et al., 2013; Mataix‐Cols et al., 2014; Scheeringa & Weems, 2014; Storch et al., 2010) have often included a wide age range, spanning children and adolescents. DCS may have differential effectiveness across age due to neurocognitive maturation. Animal and human studies suggest that extinction learning and, more specifically, extinction retention, may be impaired during adolescence relative to childhood (and adulthood) (McCallum, Kim, & Richardson, 2010) most likely due to an immature PFC control relative to amygdala‐based reactivity (Skinner & Zimmer‐Gembeck, 2016; chapter 5). Therefore, DCS might facilitate fear extinction retention by enhancing activity in the amygdala or the prefrontal cortex during extinction. One fMRI study in adults with snake phobia found that DCS produced long‐lasting changes to prefrontal activity (Nave, Tolin, & Stevens, 2012). Thus, DCS may be beneficial for adolescents by increasing prefrontal control over amygdala‐based reactivity during exposure to feared stimuli during OST. On the other hand, increasing prefrontal control with DCS during childhood, prior to the neuro‐developmentally sensitive window of adolescence, might produce stronger outcomes from OST. To date, there have been no studies of DCS augmentation of exposure therapy in youth which have examined differences between children and adolescents.

Within‐session fear reduction is a second factor which has recently been found to moderate DCS outcomes (Smits et al., 2013). Smits et al. (2013) re‐analyzed data from a prior study for acrophobia in adults which failed to find a significant effect for DCS (Tart et al., 2013) and found that the effect of DCS on clinical improvement was significantly moderated by the level of fear experienced just before concluding the previous exposure sessions. Patients receiving DCS exhibited significantly greater improvement in symptoms relative to patients who received placebo when fear was low at the end of the exposure; however, higher end of session fear was associated with less improvement in the following session compared to those who received placebo. These findings suggest that DCS may augment the direction of emotional learning taking place during exposure therapy, that is, learning associated with both reduction of fear and enhancement of fear. To date, no studies have examined the degree of fear reduction as a moderator of outcomes following OST in youth.

### 1.2 The present study

The present study was a preliminary randomized, placebo‐controlled double‐blind pilot trial of DCS‐augmented OST for children and adolescents (7–14 years) with specific phobia, with follow‐up at 1 week, 1 month, and 3 months following treatment. The primary aim was to examine the preliminary efficacy of DCS‐augmented OST in youth with specific phobias at post‐treatment and follow‐up assessments. It was hypothesized that DCS would be associated with greater improvements on primary outcomes of diagnostic severity, symptom severity, and functional impairment, as well as greater improvements on secondary outcomes of self‐reported and parent‐reported symptoms of anxiety relative to a placebo control condition.

Secondly, this study examines the potential moderating role of age and within‐session fear reduction on DCS outcomes in exploratory analyses to generate hypotheses for larger, future randomized controlled trials. The first moderator was age, whereby the effects of OST with DCS were estimated for children and compared to the effects for adolescents. In the absence of prior research, we expected on the basis that adolescence is a window of neurodevelopmental vulnerability (Johnson & Casey, 2015) that DCS effects may be attenuated for adolescents (aged ≥11 years) versus children (aged <11 years). Adolescents were defined as age 11 and above given the evidence that the majority of both boys and girls in Australia show physical signs of puberty by this age (Edwards, 2014).

Within‐session fear reduction on DCS augmentation (i.e., defined as percent reduction in subjective units of distress across the session) was used as the second moderating factor. Based on prior adult studies (Smits et al., 2013), we hypothesized that the therapeutic effects of DCS‐augmented OST would be stronger for those with the greatest reduction of fear during the OST, relative to others with lower reduction of fear.

## 2 METHOD

### 2.1 Participants

Children and adolescents were recruited via advertisements in school newsletters, referrals from health professionals, and media announcements. To be eligible to participate, youth were required to meet diagnostic criteria for a specific phobia, be aged between 7 and 17 years, have at least one parent willing to engage in the treatment, and if taking psychotropic medication be stabilized on their current dose for at least 12 weeks. Exclusion criteria included a nonanxiety primary diagnosis, autism spectrum disorders, intellectual impairment, significant learning difficulties, organic brain injury, psychosis, active suicidality, concurrent psychotherapy, taking medications contraindicated with DCS, pregnancy, and history of seizure or other serious medical condition.

**Figure 1:**
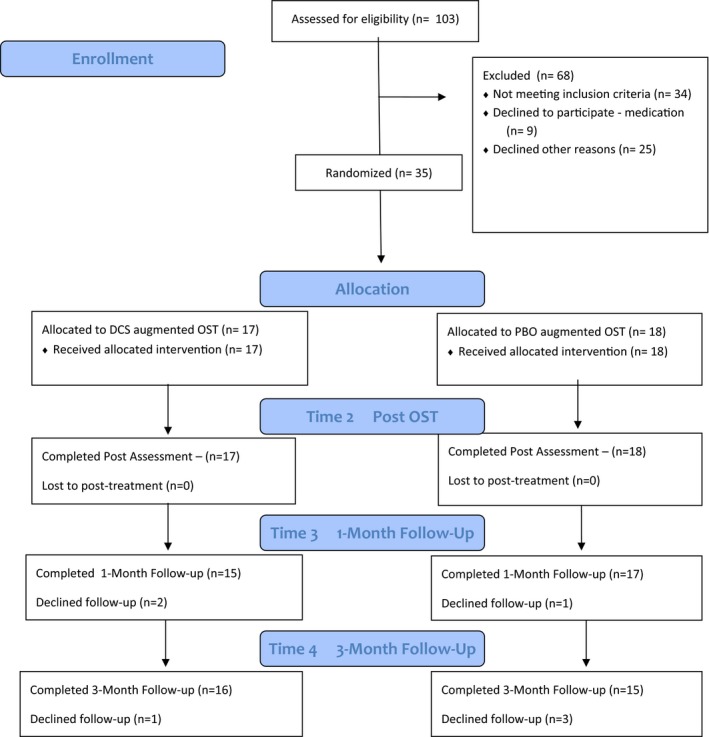
Flow of participants through trial. DCS, D‐Cycloserine; PBO, placebo; OST, one‐session treatment

One hundred and three families completed an initial telephone screen. Of those screened, 34 did not meet eligibility criteria (e.g., autism spectrum disorder, learning difficulties), nine families declined to be involved due to concerns regarding taking medication or having a blood test, and 25 families did not enroll in the trial due to other reasons (e.g., their child was reported to be improving or unable to be recontacted; see Figure 1). Thirty‐five children (71% male, *n *= 25) aged 7–14 years (*M *=* *10.43, *SD*=2.11 years) participated in the study. Sample size was estimated based upon power calculations using published effect sizes from adult studies (Kushner et al., 2007; Wilhelm et al., 2008). There were 18 children aged 7–10 years (*M* = 8.72, *SD* = 0.253) and 17 adolescents aged 11–14 years (*M* = 12.24, *SD* = 0.30). All participants had a primary anxiety disorder diagnosis, with 83% (*n *= 29) presenting with a primary phobia. The sample was highly comorbid, with youth meeting criteria on average for 3.5 diagnoses (*SD* = 1.77). Table 1 and 2 presents participant characteristics and diagnostic information.

**Table 1:** Participant characteristics by treatment condition and completer status

	Overall Sample *N *= 35	DCS + OST *N *= 17	PBO + OST *N *= 18	Completer *N *= 29	Noncompleter *N *= 6
Age—Mean (SD)	10.43 (2.11)	10.00 (2.10)	10.83 (2.09)	10.62 (1.86)	9.50 (3.14)
Gender—% Male (*n*)	71 (25)	76 (13)	67 (12)	72 (21)	67 (4)
Ethnicity—% Caucasian (*n*)	100 (35)	100 (17)	100 (18)	100 (29)	100 (6)
Marital Status —% Married (*n*)	88.5 (31)	94 (16)	72 (13)	83 (24)	83 (5)
Household Income—%					
Above 80,000 (*n*)	48.5 (17)	56 (10)	44 (8)	45 (13)	83 (5)
Psychotropic medication % (*n*) (i.e., clonidine and fluvoxamine)	5.7 (2)	0 (0)	5.7 (2)	5.7 (2)	0 (0)
Mean (SD) CSR Phobia (1–8)	6.31 (0.79)	6.18 (0.73)	6.44 (0.86)	6.34 (0.81)	6.17 (0.75)
Mean (SD) CGI‐S (1–7)	5.60 (0.69)	5.35 (0.61)	5.83 (0.71)	5.66 (0.72)	5.33 (0.52)
Mean (SD) CGAS (0–100)	57.43 (6.9)	56.39 (7.8)	58.53 (5.8)	56.55 (6.69)	61.67 (6.83)

**Table 2:** Pretreatment primary diagnosis and comorbid diagnoses

	Primary	Secondary	Tertiary	Fourth
Specific Phobia	83% (*n *= 29)	43% (*n *= 15)	23% (*n *= 8)	20% (*n *= 7)
Specific phobia—animal	40% (*n *= 14)	17.1% (*n *= 6)	2.9% (*n *= 1)	5.7% (*n *= 2)
Specific phobia—natural environment	28.5% (*n *= 10)	17.1% (*n *= 6)	5.7% (*n *= 2)	2.8% (*n *= 1)
Specific phobia—situational	0% (*n *= 0)	0% (*n *= 0)	0% (*n *= 0)	0% (*n *= 0)
Specific phobia—blood, injection, injury (BII)	1% (*n *= 5)	5.5% (*n *= 2)	8.6% (*n *= 3)	5.7% (*n *= 2)
Specific phobia—other a	11.4% (*n *= 4)	2.8% (*n *= 1)	5.7% (*n *= 2)	5.7% (*n *= 2)
Generalized anxiety disorder	2.8% (*n *= 1)	34.3% (*n *= 12)	20% (*n *= 7)	0% (*n *= 0)
Social phobia	2.8% (*n *= 1)	5.7% (*n *= 2)	17.1% (*n *= 6)	11.4% (*n *= 4)
Separation anxiety disorder	8.5% (*n *= 3)	2.8% (*n *= 1)	5.7% (*n *= 2)	5.7% (*n *= 2)
Obsessive‐compulsive disorder	0% (*n *= 0)	0% (*n *= 0)	0% (*n *= 0)	5.7% (*n *= 2)
Major depressive disorder	0% (*n *= 0)	2.8% (*n *= 1)	0% (*n *= 0)	0% (*n *= 0)
Attention Deficit/Hyperactivity	2.8% (*n *= 1)	5.7% (*n *= 2)	8.6% (*n *= 3)	2.8% (*n *= 1)
Total	100% (*N *= 35)	94% (*N *= 33)	74.3% (*N *= 26)	46% (*N *= 16)

a Specific Phobia—other = Vomit phobia, doctor/ dentist phobia and loud noises.

### 2.2 Power and design

The sample size for this pilot trial was estimated based on power calculations informed by our prior study of DCS‐augmented CBT for pediatric OCD (Farrell et al., 2013), whereby we reported significant time X treatment condition interactions with effect sizes ranging from η^*2*^ = 0.18 to η^*2*^ = 0.33. It was estimated that a sample of *n *= 12 per cell with 2 groups, and 4 time points, would have 95% power to detect an effect size of *F* = 0.46, and η^*2*^ = 0.18. Thus, an overall *n *= 35 was deemed sufficient for the exploratory (e.g., hypothesis generating) nature of this work. Given the pilot nature of this study, the sample size was not estimated based on power analysis to detect between‐group differences at study end points.

Children were randomly assigned, using a computer‐generated list of randomly permuted blocks of pairs with an allocation of 1:1, to either DCS + OST (*n* = 17) or PBO + OST (*n* = 18). The study pharmacist (ET) managed the blinding. All other investigators were blind to treatment condition, as were assessors, therapists, and participants. Pills were compounded to be identical in size and color and were dispensed by the study pharmacist (ET), immediately prior to the OST. Immediately before the commencement of the OST, the therapist gave the child the pill and observed them ingest it. A differential dose of DCS was used (35 mg or 70 mg) dependent on child weight (i.e., <45 kg = 35 mg, and >46 kg = 70 mg) in line with previous studies (Farrell et al., 2013). Children were assessed prior to OST and 1‐week, 1 month, and 3 months following OST.

### 2.3 Measures

#### 2.3.1 Primary outcome measures: child diagnostic status, severity, and functioning

*Anxiety Disorders Interview Schedule for Children—Child and Parent version* (ADIS‐IV; Silverman & Albano, 1996). The ADIS‐IV‐C/P is a psychometrically robust (Silverman, Saavedra, & Pina, 2001; Wood, Piacentini, Bergman, McCracken, & Barrios, 2002), semi‐structured interview, specifically developed to diagnose anxiety, mood, and other disorders in children aged 6–17 years. Independent blind assessors were used for each assessment point. Assessors were clinical psychology postgraduate research students who were trained to reliability to complete interviews by firstly attending a workshop on the administration of the ADIS‐C/P, followed by observing a number of interviews conducted by expert assessors (*n *= 3 to 5 interviews), and then completing interviews with supervision of each interview by the first author (LJF). Interrater reliability was conducted across 20% of the recorded interviews by independent raters, with results indicating excellent reliability (primary diagnosis κ = 0.94; secondary diagnosis κ = 0.88; tertiary diagnosis κ = 0.86).

*Clinical Global Impressions Scale* (CGI; Leon et al., 1993). The CGI is an extensively used, clinician‐rated scale designed to assess (1) severity of psychopathology (CGI‐Severity; CGI‐S) and (2) change following treatment (CGI‐Improvement; CGI‐I). The CGI‐S is rated on a 7‐point Likert‐type scale from 1 (*normal*) to 7 (*among the most extremely ill*), whereas the CGI‐I rates improvement from 1 (*very much improved*) to 7 (*very much worse*). The same rater who completed the ADIS‐P completed the CGI. The CGI has been found to have sound psychometric properties (Leon et al., 1993).

*Children's Global Assessment Scale* (CGAS, Shaffer et al., 1983). The Children's Global Assessment Scale (CGAS) is well‐established measure of youth's overall functioning and level of impairment. It is clinician rated with scores ranging from 1 (*needs constant supervision*) to 100 (*superior functioning*).

#### 2.3.2 Secondary outcome measures: child symptoms and within‐session fear reduction

*Spence Children's Anxiety Scale Child and Parent Versions* (Spence, 1998). The SCAS‐C/P is a measure of anxiety symptoms in children aged 7–18 years. It has well‐established reliability and validity (Nauta et al., 2004; Spence, 1998). The internal consistency in the current study was excellent, with Cronbach's alpha 0.91 for SCAS‐P and 0.89 for the SCAS‐C.

*Subjective Units of Distress (SUDs) Ratings within OST*. During the OST, children rated their subjective units of distress (SUDs) on a 9‐point Likert scale from 0 (not scared at all) to 8 (very, very scared). Ratings were obtained from at least three exposure tasks per hour and were taken immediately before an exposure task, intermittently throughout the exposure task, and at the end of the exposure task. An exposure task was deemed completed when the child reported minimal anxiety (SUDs rating of 0 or 1), or when at least a 50% reduction in fear had occurred. A mean percentage reduction in SUD ratings was calculated for the overall session.

### 2.4 Procedure

#### 2.4.1 Pretreatment

Following approval by the university Human Subjects Review Committee, the trial was registered with the Australian and New Zealand Clinical Trials Registry (no. ACTRN12612000420842). Upon initial contact, parents completed a brief telephone interview to assess their child's eligibility. Eligible, consenting families completed a parent diagnostic interview (ADIS‐IV‐P) via the telephone, followed by assessment in the clinic. In the clinic, the child was administered the child diagnostic interview (ADIS‐IV‐C), and both children and their parents completed self‐report measures. CGI and CGAS ratings are made by the independent assessors following both the parent and child diagnostic interviews, taking into account the child's full clinical presentation. The assessors then reported the results of the standardized diagnostic interviews, including CSR ratings for each diagnosis, which were reviewed and moderated at a team diagnostic consensus meeting overseen by the first author (LJF). CGI and CGAS ratings were also reviewed and moderated by the team to ensure reliable ratings. A final team consensus rating was determined based all the relevant diagnostic and clinical information. A consultant psychiatrist (VG, NC, HM) reviewed children's laboratory tests (e.g., complete blood count, metabolic panel, and pregnancy) and provided a prescription for study medication (i.e., DCS). Children then proceeded to treatment.

#### 2.4.2 One‐session treatment

Participants completed an OST session for their primary phobia diagnosis. The treatment was manualized (Öst & Ollendick, 2001), was 3 hr in duration, and involved exposure therapy along with cognitive challenges, participant modeling, contingency management, and psychoeducation (see Davis, Ollendick, Reuther, & Muson, 2012 for a detailed description). The treatment was modified from the original manual in two minor ways: Parents were actively involved in the treatment (see below), and a maintenance program following the OST was incorporated which consisted of brief phone calls for three weeks following treatment to monitor progress and encourage ongoing exposure practice. Participants completed a range of exposure tasks during OST, with at least three phobic objects or stimuli introduced over the course of the session (e.g., small, medium, and large dog). All children completed their OST. At the commencement of the session, parents were provided with psychoeducation handouts, covering phobias, the principles of exposure therapy, and contingency management strategies. Parents were invited to participate in the last half hour of the OST where they reviewed the child's progress, asked any questions and discussed the importance of continued practice. Collaboratively, the therapist and family decided upon home exposure practice. Parents received brief telephone calls once per week for 4 weeks to review progress. The OST was conducted by clinically trained therapists (postgraduate students), who attended an intensive 2‐day workshop regarding OST, delivered by the last author (THO). Following training, all therapists observed an OST session with an expert therapist in OST prior to conducting a session. All OST sessions were planned and supervised by the first author (LJF) to ensure standardization in the delivery of treatment.

### 2.5 Data analysis

Baseline differences across treatment conditions (DCS versus PBO) and completer status (completer versus noncompleter) were analyzed using independent *t* tests for continuous variables and chi‐square tests for categorical variables.

Linear mixed‐effects models were used in order to examine the overall treatment effects for primary outcome measures (CSR, CGI‐S, CGAS), with one model fitted for each outcome. Time of assessment (four levels: 1 = pretreatment, 2 =  post‐treatment, 3 = 1‐month follow‐up, 4 = 3‐month follow‐up) was the within‐subject effect, and treatment condition (DCS vs. PBO) was the between‐subject effect. A quadratic pattern of change (time^2^) was also tested in each model.

We used similar linear mixed‐effects models to test whether age moderated treatment effects on the primary outcome measures. Age groups were defined as *children* aged 7–10 years versus *adolescents* aged from 11 to 14 years. Two 2‐way interactions and one 3‐way interaction were entered into the models to test moderation (i.e., age x condition, age x time, and age x condition x time).

In these linear mixed‐effects models, we used the Monte Carlo Markov chain (MCMC) scheme to estimate model parameters. We used the library MCMCglmm in R (R Core Team, 2015 2014) to do this estimation. Posterior estimates are based on 100,000 iterations completed after a 50,000 iteration burn‐in and thinned at a rate of 10. Vague conjugate priors were used for the coefficients and variance. Output from the MCMC analyses was used to calculate p‐values for treatment and other grouping comparisons for all models. These were calculated as the posterior probability of a specific effect being greater or less than another effect. In this article, we refer to a significant effect as one where there is a less than 5% chance that the posterior probability for the coefficient (i.e., effect size) included zero. Thus, for all Bayesian analyses, results are described as “significant” at Bayesian p‐value <0.05 (p‐values are not reported in text in line with standard reporting for these analyses and to ease interpretation of the analyses, except where the Bayesian p‐values were marginally significant and >0.05). Posterior distributions are described by their mean, and credible intervals are given in brackets. In the case of descriptive statistics using t tests and chi‐square analyses, we report the actual frequentist p‐value (in line with standard reporting for these statistics). Finally, the effect of age group, treatment, and overall within‐session mean SUDs reduction on CGAS was tested using a Bayesian linear regression with noninformative priors.

## 3 RESULTS

### 3.1 Baseline group differences

Children in the two treatment conditions (intent‐to‐treat sample) did not differ on any demographic data (see Table 1), on primary outcome measures, or on self‐reported anxiety at baseline. There was no age by treatment condition differences at baseline on any of the primary or secondary outcome measures. Children who completed follow‐up assessments (*n *= 29) did not differ from those who did not (*n *= 6) across demographic or symptom measures at baseline or at post‐treatment.

### 3.2 Treatment effects—primary outcome measures

Table 3 presents the modeled means (and their lower and upper credible intervals) for each treatment condition for CSR, CGI‐S, and CGAS. Overall, there was no significant effect of treatment condition on CSR, CGI‐S, or CGAS, nor was there a treatment condition x time interaction on any of these measures for the overall sample. As such, treatment condition was removed from each model and the data were re‐analyzed with only the effect of time. In each of these models, symptoms declined over time. CSR decreased significantly from 6.24 (5.68–6.80) at pretreatment to 3.61 (3.05–4.18) at post‐treatment. At 1 month, CSR had decreased to 2.45 (1.70–3.19). At 3 months post‐treatment, the estimate was 2.45 (1.69–3.77). *Similarly,* CGI‐S and CGAS scores significantly declined over time.

**Table 3:** Modeled means and 95% lower and upper credible intervals (LCI, UCI) for treatment condition, over time, and across age groups

Variable	DCS Mean (LCI, UCI)			PBO Mean (LCI, UCI)		
	Overall	Child	Adolescent	Overall	Child	Adolescent
CSR						
Pre	6.18 (5.58, 6.76)	6.09 (5.37, 7.23)	6.34 (5.31, 7.34)	6.44 (5.90, 7.00)	6.14 (5.58, 7.23)	6.46 (5.31, 7.34)
Post	3.65 (2.87, 4.41)	3.18 (2.22, 4.11)	4.50 (3.20, 5.77)	3.17 (2.45, 3.89)	3.89 (2.81, 4.99)	2.72 (1.77, 3.67)
1 month	2.30 (1.36, 3.25)	1.96 (0.80, 3.09)	2.92 (1.28, 4.53)	3.06 (2.21, 3.92)	3.97 (2.63, 5.33)	2.53 (1.43, 3.66)
3 months	2.72 (1.65, 3.77)	2.47 (1.14, 3.80)	3.16 (1.38, 4.90)	2.61 (1.63, 3.58)	2.81 (1.30, 4.40)	2.45 (1.13, 3.78)
CGI						
Pre	5.35 (4.90, 5.83)	5.19 (4.60, 5.76)	5.67 (4.88, 6.45)	5.83 (5.41, 6.24)	5.84 (5.22, 6.48)	5.82 (5.25, 6.40)
Post	2.95 (2.40, 3.50)	2.64 (1.95, 3.33)	3.49 (2.55, 4.44)	2.45 (1.92, 2.98)	2.74 (1.94, 3.53)	2.28 (1.58, 2.99)
1 month	2.01 (1.33, 2.67)	1.63 (0.80, 2.46)	2.72 (1.55, 3.89)	2.46 (1.85, 3.07)	3.29 (2.29, 4.24)	2.00 (1.18, 2.79)
3 months	2.36 (1.60, 3.09)	2.01 (1.06, 2.94)	3.00 (1.73, 4.26)	2.06 (1.34, 2.77)	2.16 (1.04, 3.31)	1.99 (1.04, 2.93)
CGAS						
Pre	58.51 (55.11, 61.92)	58.64 (54.40, 62.94)	58.33 (52.48, 64.28)	56.43 (53.33, 59.60)	59.43 (54.67, 64.20)	54.53 (50.24, 58.85)
Post	69.15 (64.85, 73.35)	71.39 (66.27, 76.66)	65.01 (57.82, 71.97)	69.06 (65.06, 73.07)	65.44 (59.37, 71.46)	71.41 (66.28, 76.65)
1 month	73.93 (68.80, 79.00)	76.15 (70.27, 82.07)	70.00 (61.71, 78.28)	72.10 (67.46, 76.79)	69.36 (62.41, 76.32)	73.67 (67.82, 79.48)
3 months	71.06 (65.58, 76.60)	77.44 (71.05, 83.86)	59.99 (51.56, 68.41)	75.17 (70.07, 80.43)	72.73 (64.94, 80.58)	76.55 (70.09, 83.13)
SCAS‐P						
Pre	32.25 (26.80, 37.63)	34.29 (27.73–40.68)	28.77 (19.07–38.40)	29.29 (23.58, 35.15)	32.42 (23.22–41.58)	28.04 (20.10–36.12)
Post	25.76 (20.92, 30.56)	26.00 (20.78–31.44)	24.49 (16.94–31.84)	24.28 (19.20, 29.40)	30.74 (22.89–38.40)	20.76 (14.91–26.87)
1 month	19.28 (14.08, 24.60)	17.71 (12.04–23.69)	20.20 (12.50–27.62)	19.28 (13.18, 25.34)	29.05 (19.71–38.06)	13.48 (6.39–20.78)
3 months	12.79 (6.12, 19.52)	9.42 (1.81–17.3)	15.92 (6.00–25.47)	14.27 (6.00, 22.21)	27.37 (14.83–39.84)	6.20 (−4.35–16.68)

Mean values are significant when the credible interval does not contain 0.

### 3.3 Treatment effects by age—primary outcome measures

#### 3.3.1 CSR

**Figure 2:**
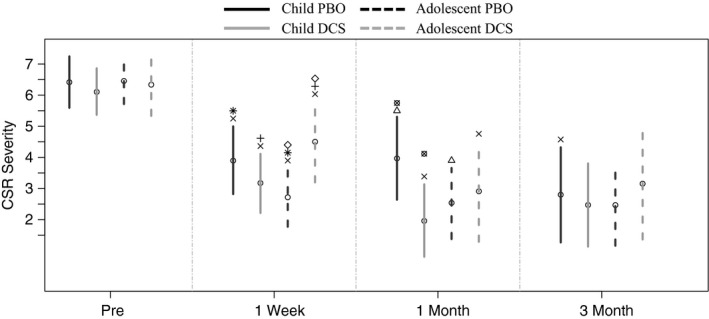
Effects of treatment condition over time, and across children versus adolescent on CSR. x  =  Significant effect over time from previous assessment; all other symbols represent between‐group differences (conditions with same symbol) within each time point. CSR = Clinician severity ratings of treated phobia diagnosis

When age was examined as a moderator of treatment group effects on CSR, there were significant differences between treatment conditions. Both children and adolescents declined in CSR across conditions from pre‐ to post‐treatment. Further, both children and adolescents in the DCS condition had a significant decline in CSR from post‐treatment to 1 month relative to those in PBO (see Figure 2). Children in the PBO condition were the only group who had a significant decline in CSR from 1‐month to 3‐month follow‐up. When groups were compared at post‐treatment, adolescents in the DCS condition had a significantly higher average CSR than adolescents in PBO, but children in DCS and PBO groups did not differ. At 1‐month follow‐up, children in the DCS condition were significantly lower in CSR relative to children in the PBO condition, but adolescents in both DCS and PBO did not differ. At 3‐month follow‐up, there were no significant between‐group differences on CSR ratings.

#### 3.3.2 CGI‐S

When age was examined as a moderator of treatment effects on CGI‐S, children and adolescents in both the DCS and PBO groups had a significant decrease from pretreatment to post‐treatment. Children in the DCS condition had a further significant decline from post‐treatment to 1 month, and adolescents in the DCS condition had a marginal decline. Finally, children in the PBO condition declined significantly from 1‐month to 3‐month follow‐up.

When treatment groups were compared at post‐treatment within each age group, adolescents receiving DCS were significantly higher on CGI ratings than adolescents in the PBO condition, whereas children in the DCS and PBO conditions did not differ. When treatment groups were compared at 1‐month follow‐up by age group, children in the DCS condition were significantly lower on CGI‐severity relative to children in the PBO condition; however, for adolescents, DCS and PBO did not differ. At 3 months following treatment, there were no significant between‐group differences on CGI ratings.

#### 3.3.3 CGAS

**Figure 3:**
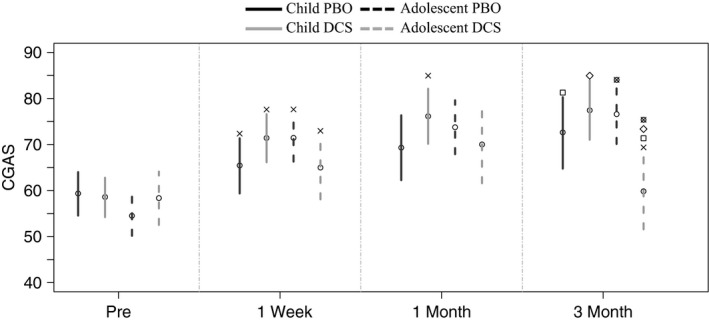
Effects of treatment condition over time, and across children versus adolescent on Clinical Global Assessment Scale. x = Significant effect over time from previous assessment; all other symbols represent between‐group differences (conditions with same symbol) within each time point. CGAS = Clinical Global Assessment Scale; Child PBO, Child DCS

When age was examined as a moderator, children and adolescents in both the DCS and PBO groups showed significant improvements from pretreatment to post‐treatment (see Figure 3). Children in the DCS condition had a further significant improvement from post‐treatment to 1 month. Adolescents in the DCS condition had a further significant improvement from 1‐month to 3‐month follow‐up.

At post‐treatment and 1‐month follow‐up, there were no significant differences in CGAS scores between treatment conditions for children or adolescents. However, at 3‐month follow‐up, adolescents in the DCS condition had significantly lower CGAS scores than adolescents in the PBO condition, children in the DCS condition, and children in PBO (see Figure 3).

### 3.4 Treatment effects—secondary self‐reported outcome measures

#### 3.4.1 SCAS‐C

There was no significant effect of treatment condition or age group for self‐reported anxiety symptoms. There was also no significant interaction of treatment condition or age group with time. However, there was a significant decrease in SCAS‐C reports from pretreatment 30.9 (26.2–35.5) to post‐treatment 24.5 (20.3–28.6). Also, there was a further significant decline from post‐treatment to 1‐month follow‐up to 18.0 (13.6–22.7). There was no significant change from 1‐month to 3‐month follow‐up of 11.6 (6.1–17.3), which may be due to the reduced numbers resulting in increased variability.

#### 3.4.2 SCAS‐P

**Figure 4:**
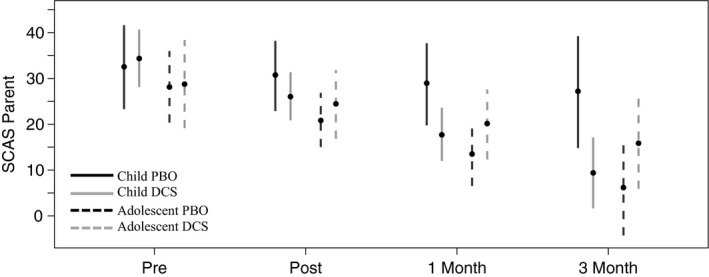
Effects of treatment condition over time, and across children versus adolescent on SCAS‐P ratings. SCAS‐P, Spence Child Anxiety Scale ‐ Parent report

Parent‐reported anxiety significantly declined from pretreatment to post‐treatment in all groups, with the exception of children in the PBO condition (see Figure 4). Children in the DCS condition had a further significant decline from post‐treatment to 1‐month, and 1‐month to 3‐month follow‐up. Adolescents in both the DCS and PBO conditions declined significantly from post‐treatment to 1‐month, and from 1‐month to 3‐month follow‐up.

SCAS‐P scores also differed between treatment conditions for children. At 1‐month follow‐up (see Figure 4), children in the DCS condition had significantly lower SCAS‐P scores than children in the PBO condition. At 3‐month follow‐up, children in the DCS condition also had significantly lower SCAS‐P scores than children in the PBO condition. No difference between DCS and PBO at 1‐month and 3‐month follow‐up for adolescents was observed.

### 3.5 Effects of within‐session fear reduction

**Figure 5:**
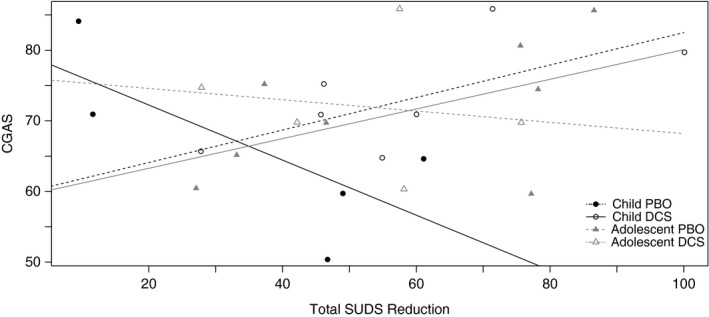
Effects of within‐session SUDs reduction on treatment condition for children versus adolescents, in predicting CGAS ratings at 1‐month follow‐up. CGAS, Clinical Global Assessment Scale

In order to examine the moderating effects of within‐session SUDs reduction on treatment effects across time and group, a Bayesian linear regression model was estimated to predict CGAS ratings at 1‐month follow‐up. The results of the analysis were consistent with our hypotheses, but all associations were marginal. When within‐session SUDs reduction was higher (i.e., greater SUDs decline), children in the DCS condition were functioning better (had a significantly higher CGAS) at 1‐month follow‐up, relative to children in the PBO condition (*p *=* *.07; see Figure 5, Table 4). The opposite was found for adolescents; adolescents in the DCS condition with a higher reduction in SUDs were functioning worse at 1‐month follow‐up, relative to adolescents in the PBO condition (*p *=* *.07).

**Table 4:** Coefficients and credible intervals (CI) for within‐session SUDs in predicting CGAS ratings over time as a function of treatment condition and age group (child or adolescent)

Treatment condition and age group	Intercept Mean (lower and upper CI)	Slope Mean (lower and upper CI)
Placebo Child	80.04 (64.21–95.76)	−0.39 (−0.77 to 0.01)
DCS Child	59.51 (39.81–79.05)	0.23 (−0.09 to 0.55)
Placebo Adolescent	59.09 (41.56–76.51)	0.21 (−0.07 to 0.49)
DCS Adolescent	76.21 (49.25–103.12)	−0.08 (−0.57 to 0.41)

Mean values are significant when the credible interval does not contain 0.

### 3.6 Diagnostic status across time

Table 5 presents the diagnostic data for participants across time and treatment condition, including for the completer sample, as well as the intent‐to‐treat sample (last observation carried forward). There were no significant differences across DCS or PBO conditions on percent of participants free from their treated phobia diagnosis at any time point, or on the percentage of children free from any diagnosis at each time point. Likewise, there were no significant differences between treatment conditions at each time point on the overall number of diagnoses.

**Table 5:** Diagnostic status across treatment conditions and time for completer sample and intent‐to‐treat sample

	Pretreatment			Post‐Treatment (1 week)			1 Month Follow‐Up			3‐Month Follow‐Up		
	DCS	PBO	t test	DCS	PBO	Chi‐square / t test	DCS	PBO	Chi‐square / t test	DCS	PBO	Chi‐square / t test
Completer												
% (*n*) Phobia Diagnosis Free	–	–		29 (4)	61 (11)	χ^2^ (1) = 3.54, *p* = .06	73 (11)	59 (10)	χ^2^ (1) = 0.744, *p *= .31	50 (8)	73 (11)	χ^2^ (1) = 1.77, *p *= .17
% (*n*) Free of all diagnoses	–	–		18 (3)	27 (5)	χ^2^ (1) = 0.008, *p *= .62	35 (6)	39 (7)	χ^2^ (1) = 0.005, *p *= .62	24 (4)	39 (7)	χ^2^ (1) = 0.008, *p *= .31
Mean No. of Diagnoses (SD)	3.88 (1.73)	3.33 (1.82)	*t*(33) = −0.92, *p *= .37	1.58 (1.06)	1.66 (1.68)	*t* (33) = 0.16, *p *= .87	0.80 (0.86)	1.06 (1.19)	*t* (30) = 0.69, *p *= .49	1.4 (1.15)	1.33 (1.55)	*t* (29) = −0.62, *p *= 0.54
Intent‐to‐treat												
% (*n*) Phobia Diagnosis Free	–	–		29 (5)	61 (11)	χ^2^ (1) = 3.54, *p *= .06	65 (11)	61 (11)	χ^2^ (1) = 0.048, *p *= .55	53 (9)	78 (14)	χ^2^ (1) = 2.39, *p *= .12
% (*n*) Free of all diagnoses	–	–		18 (3)	28 (5)	χ^2^ (1) = 0.008, *p *= .62	35 (6)	39 (7)	χ^2^ (1) = 0.048, *p *= .55	24 (3)	44 (9)	χ^2^ (1) = 4.70, *p *= .09
Mean No. of Diagnoses (SD)	3.88 (1.73)	3.33 (1.82)	*t*(33) = −0.92, *p *= .37	1.58 (1.06)	1.66 (1.68)	*t* (33) = 0.16, *p *= .87	0.88 (0.85)	1.16 (1.29)	*t* (33) = 0.76, *p *= .45	1.70 (1.53)	0.94 (1.10)	*t* (33) = −1.69, *p *= .10

## 4 DISCUSSION

The primary aim of the current pilot study was to examine the augmenting effects of DCS on phobia severity following an intensive OST for youth. A secondary hypothesis generating aim was to explore both patient‐level and therapy‐level variables which may be associated with moderating the effects of DCS augmentation, specifically, the effects of age and within‐session fear reduction.

Contrary to hypotheses, when examining the overall sample, there were no significant augmenting effects of DCS on OST on any of the primary or secondary outcome variables. This finding is in line with other studies that found no overall benefits of DCS on outcomes among youth, including those with mixed anxiety disorders (Rapee et al., 2016), as well as PTSD (Scheeringa & Weems, 2014) and OCD (Mataix‐Cols et al., 2014). When examining secondary analyses of the effects of DCS on age over time, the findings provide initial support for positive DCS augmenting effects among children across outcome measures (CSR, CGI‐S, SCAS‐P) at 1‐month follow‐up, relative to children in the PBO condition. Further, children in the DCS condition showed significantly greater improvement from post‐treatment to 1 month following treatment on CGAS ratings. For adolescents however, there were significant benefits in the PBO condition relative to DCS at post‐treatment (CSR, CGI‐S), and moreover, youth in the DCS condition experienced significantly poorer global functioning at 3‐month follow‐up, relative to all other treatment conditions.

These results offer preliminary data that age may moderate response to DCS‐augmented exposure therapy for young people with specific phobias. Findings from basic science suggest that adolescence is a period marked by impaired extinction learning relative to younger and older developmental stages (McCallum et al., 2010; Kim & Richardson, 2010) due to developmental differences in neural mechanisms underlying extinction during adolescence (see Baker & Richardson, 2015). However, our results do not suggest impairment in extinction learning among adolescents, with those in the PBO condition doing as well as children who received DCS, and significantly better than children in the PBO condition. Indeed, human research has generally shown that age is not a significant predictor of outcome from exposure therapy for child anxiety disorders generally (see Kendall & Peterman, 2015), or for child phobias more specifically (Ollendick & Davis, 2013). However, those adolescents who received DCS exhibited a significantly poorer response relative to adolescents in PBO. One explanation is that activation of NMDA in the amygdala via DCS interferes with neural activation associated with extinction learning during adolescence. Replication of the present findings in larger samples is required before firm conclusions can be drawn, and further research on underlying developmental differences is warranted.

Children on the other hand demonstrated improved outcomes in the DCS condition at 1 month following OST relative to the PBO condition on numerous outcomes and relative to youth in the DCS condition. This finding is consistent with our previous trial for difficult‐to‐treat pediatric OCD (Farrell et al., 2013), whereby DCS was associated with greater improvement from post‐treatment to 1‐month follow‐up relative to the PBO condition. Similarly, this study demonstrated augmenting effects appear to be associated with accelerated gains immediately following treatments and up to 1 month following treatment; however, by 3‐month follow‐up there were few differences between treatment conditions. This finding is consistent with the wider literature on DCS augmentation, which suggests that DCS effects are time‐limited. One possibility for future research is to explore the outcome of targeted dosing of exposure and DCS at 1 month following treatment, in order to further leverage DCS effects, and perhaps provide longer‐term benefits for patients.

Our findings also provide preliminary support for recent research (i.e., Smits et al., 2013) suggesting that the quality of exposure experiences likely moderates the augmenting effects of DCS. For example, if DCS enhances learning during exposure, then it potentially enhances both good and suboptimal learning. Our findings are consistent with Smits et al. (2013), whereby DCS augmentation was marginally associated with improved functioning at 1‐month follow‐up for children who experienced a greater reduction of fear (SUDs) during the OST session. For children with less reduction in fear during OST, their functioning was worse in the DCS condition relative to the PBO. This finding may be of high clinical relevance if replicated in a larger sample. If DCS is associated with augmenting successful exposure, but attenuates poor exposure outcomes, then postsession tailored dosing of DCS following successful exposure therapy may be an optimal model for clinical practice. To date, there are no controlled trials of the effects of differential timing of dosing of DCS (pre‐ versus postsession dosing), nor controlled trials specifically examining a tailored dosing paradigm—research that would further inform practical guidelines for DCS augmentation.

In addition to a small sample size, this study has other limitations. Firstly, the age range in the current study was narrower than we anticipated. While inclusion was defined as 7–17 years, recruitment, however, only resulted in a sample that extended to 14 years of age. Thus, further research should address age as a moderator of response to DCS‐augmented OST using a broader age range. While this study employed rigorous, gold standard assessments, including blinded independent raters, we were not successful in maintaining a high rate of questionnaire completion at follow‐up due to families being required to return measures by mail, and we did not complete independent reliability for the CGI and CGAS. Our approach to managing this shortcoming, in addition to our small sample size, was to use robust analytical approaches that are particularly well suited to these issues (i.e., Bayesian modeling). Nevertheless, the findings reported here are preliminary and are limited by the small sample and multiple analyses conducted to explore study hypotheses effects. Finally, other possible confounds in the current study which may account for the null findings are the possibility of limited measurement sensitivity and, moreover, the degree to which children engaged in out of session exposure therapy. Larger trials that measure between session exposure practice, and control for this in analyses are warranted.

DCS augmentation presents a novel and interesting approach to improving exposure therapy outcomes in the treatment of anxiety disorders. This study found no overall benefit for DCS augmentation of exposure therapy for phobias among youth; however, it highlights the importance of further research aimed at identifying the precise patient‐level and therapy‐level characteristics which may moderate DCS augmenting effects. This pilot study provides preliminary evidence that child age and within‐session SUDs reduction may moderate DCS augmentation—a hypothesis which requires empirical testing in an adequately powered randomized controlled trial.

## CONFLICTS OF INTEREST

None.
